# Dissection of genomic regions underlying early seedling vigour in chickpea through genome-wide association mapping

**DOI:** 10.1186/s12870-025-07814-x

**Published:** 2025-12-05

**Authors:** Uttarayan Dasgupta, Arun Kumar M.B., Neeraj Kumar, Somsole Bharath, Shailesh Tripathi, Chellapilla Bharadwaj

**Affiliations:** 1 Division of Genetics, ICAR – Indian Agricultural Research Institute, New Delhi, 110012 India; 2 Division of Seed Science & Technology, ICAR – Indian Agricultural Research Institute, New Delhi, 110012 India; 3 AICRP On Rabi Pulses, ICAR- Indian Institute of Pulses Research, Kanpur, 208024 India

**Keywords:** Chickpea, Early seedling vigour, Genome-wide association studies (GWAS), Germination, Lateral root development, Marker-trait associations

## Abstract

**Background:**

Chickpea (*Cicer arietinum* L.) is the most important pulse in India and one of the most important worldwide. In order to increase the chickpea production area, the rainfed rice fallows are being targeted to adopt rice-chickpea cropping system. Early seedling vigour (ESV) is an important trait which enables the crop to have better germination, crop stand, utilization of residual soil moisture, faster biomass accumulation and better root growth under poor soil structure. Till date there has been no work done regarding the mapping of genomic regions controlling ESV in chickpea.

**Results:**

We conducted a genome-wide association study taking 13 traits related to ESV in a diverse panel of the reference set of ICRISAT. GWAS was conducted using FarmCPU and BLINK model and a total of 34 marker-trait associations (MTAs) were identified. We were able to identify putative 36 candidate genes linked to the MTAs such as Lateral Root Primordium 1, Auxin-Induced Protein 22D-Like, Transcription factor MYB3-like etc. Most of these genes are involved in primary and lateral root formation, development of meristem, hormone signaling and germination that ultimately regulate the seedling vigour in chickpea.

**Conclusion:**

Our findings have identified substantial genetic variability for early seedling vigour traits in chickpea. Phenotypic screening has enabled to identification of highly vigorous genotypes like ICC15567, ICC8318. We also reported novel MTAs linked to ESV traits in chickpea, which can be further validated using functional genomic studies. The findings of this study will help in further understanding of ESV as a trait and the development of early vigorous chickpea varieties in future.

**Supplementary Information:**

The online version contains supplementary material available at 10.1186/s12870-025-07814-x.

## Background

Chickpea is one of the most important pulse crops in the world with an area under cultivation of 14.096 million hectares and a production of 16.517 million tonnes. India is the largest producer of chickpea in the world contributing approximately 74% (12.27 m tonnes) of world chickpea production and accounts for 74.27% (10.47 m ha) of all cultivated areas [1]. In India, it is grown predominantly in the states of Madhya Pradesh (MP), Rajasthan, Maharashtra, Uttar Pradesh (UP), Karnataka and Andhra Pradesh (AP). Chickpea is consumed in various forms in the Indian market. These products range from immature green grains, mature desi/kabuli grains, besan (raw grain flour), roasted grains, sattu (roasted grain flour), dal, flakes, baked goods, sweets and other tertiary processed goods. The increasing use of plant-based protein products in the market (protein isolates) has raised the importance of chickpea in industries [2]. Under optimal sowing conditions, the potential yield of chickpea reaches more than 5 t/ha; however, actual chickpea production is well below the potential yield [3]. Efforts are being made to expand the area of chickpea production.

The first approach we can take to increase the production of pulses is to expand its area. We must look into other niche areas apart from irrigated-cereal and cereal-based cropping system as farmers are reluctant to adopt chickpea production in those existing cropping systems. Rain-fed rice fallows are such a situation and offer a good potential area for expanding the area under chickpea [4]. The residual soil moisture remains a constraint to successful germination and emergence in the rice fallows and as such, early seedling vigour is essential to crop establishment and survival.

According to Mahender et al. [5], seedling vigor is an agronomic trait that indicates the capacity of a seed to germinate, generate a strong seedling, and survive adverse climatic conditions. One of the important traits that influence successful crop establishment is ESV, i.e. the ability to promote rapid and uniform emergence and growth of seedlings under a range of field conditions [6]. The genotypes with high early seedling vigour have competitive advantages against abiotic stress, better crop-stand establishment, and are less susceptible to diseases that attack in the seedling stage (such as root rot and seedling blight). Further, high seedling vigour of the crop also assists in early germination, better rooting under poor soil structure, utilization of the depleted soil moisture, superior weed suppression and minimizes the loss of soil moisture due to the rapid growth of the above-ground biomass [5]. ESV is a complex trait involving a number of physiological, morphological, and biochemical processes in plants, particularly during germination and early growth, hence direct genotype selection is difficult. Germination rate, final germination percent and germination index at the seed germination stage [7], root length, shoot length, seedling weight at the early stage of seedling growth, are the morphological characters for ESV [70]. Early seedling vigour (e.g., faster root growth or more biomass) is important so that seedlings can become established in the stress-filled environment of rice fallows. ESV under different situations has been repeatedly evaluated with the seedling vigour index-I (VI-I) and seedling vigour index-II (VI-II). Both field-recorded VI-I and VI-II were positively and significantly associated with seed yield in chickpea [8].

Seedling vigour in plants is a complex genetic trait influenced by a combination of genetic and environmental conditions and their interactions [9]. Mapping for ESV has been done in crops like rice, wheat, barley, and soybean using both association mapping using diverse germplasm lines and QTL mapping using bi-parental populations and SNP markers [10–15]. In the case of chickpea, QTLs linked to traits such as Fractional Green Canopy Cover (FGCC) and Normalized Difference Vegetation Index (NDVI) have been identified which are considered important for mid-late plant vigour [16]. However, no study to date has been conducted to delineate the genetics of early seedling vigour traits in chickpea.

In our present study, we have assessed variation for early seedling vigour traits in the chickpea reference collection in a controlled environment experiment. The diversity of chickpea around the world is reflected in the chickpea reference collection and it has already been used to delineate other important traits such as drought, heat tolerance, phosphorus acquisition efficiency, phosphorus use efficiency, root-lesion nematode resistance, plant architectural, key nutritional and agronomical traits [17–22, 71]. Novel marker-trait associations (MTAs) and candidate genes were identified in our study for the ESV traits using genome-wide association analysis. The information generated from this study will be useful in deciphering the genetic control of early seedling vigor in chickpea and will have a direct application in breeding rice-fallow cropping systems with appropriate early vigorous chickpea varieties.

## Materials & methods

### Plant materials

For the present study, 230 genotypes were used for the association panel that were a part of the chickpea reference collection, consisting of 300 genotypes originating from different geographical groups. The association panel was made up of 165 desi, 57 kabuli and 8 pea-shaped genotypes. The majority of them belonged to landraces (90.4%) followed by a minor number of traditional and advanced cultivars and breeding materials. Maximum genotypes in the panel originated from India (35.2%) and Iran (28.3%), followed by Ethiopia (4.78%), Turkey (4.34%) and Afghanistan (3.91%).

The seeds of the genotypes were multiplied during Rabi 2023–2024 at experimental field of the Division of Genetics, ICAR-IARI, New Delhi (28.639549˚ N, 77.153643˚ E, 217 m). The spacing between rows was 30 cm and the spacing between plants was 10 cm. The crop was managed under normal agronomic practices. Seeds were harvested from the central plants and then stored appropriately for further phenotyping.

### Phenotyping

The screening of the chickpea accessions was constructed as a completely randomized design (CRD) with two replications and was performed in controlled conditions in the seed germination chamber of the Division of Seed Science & Technology, ICAR- Indian Agricultural Research Institute. The screening of the chickpea genotypes was conducted two months after harvesting the seeds in 2024. 50 healthy seeds from each genotype were carefully selected and taken for screening of vigour traits. The seeds were treated with Bavistin (Carbendazim 50% WP) at 2 mg/g of seeds to prevent a fungal attack during germination. Before the experiment, the seed dry weight of each accession was taken with the help of a sensitive balance. The seeds were wrapped in germination towels and placed in seed germination chamber at 20 ± 1 ˚C for 10 d. The final count and evaluation of the seedlings was done on day eleven. Germination percentage was determined by the ratio of the number of germinated seeds to the number of seeds tested. 5 healthy seedlings were drawn from each replication of each genotype to collect data on shoot length (SL), root length (RL), total seedling length (TSL), shoot fresh weight (SFW) and root fresh weight (RFW). Cotyledons and axis of the seedlings were separated and put into a hot air oven for 48 h at 50 ˚C to assess shoot dry weight (SDW), root dry weight (RDW) and remaining dry weight in cotyledons (RDWC). Use of seed reserves (USR) was calculated as, USR = seed dry weight – remaining dry weight in cotyledons (RDWC) and is expressed in mg seed^−1^. USR has been used to understand the vigour capacity of the genotypes to efficiently utilize their seed reserves [23, 72]. Subsequently, seedling vigour index I (SVI) and seedling vigour index II (SVII) were calculated as per the following formula [24, 25]:

$$\begin{aligned} \text{Seedling vigour index I}\ &=\ \mathrm{Germination}\ \% \\ & \times\ \text{Total seedling length}\ (\mathrm{TSL}\ =\ \mathrm{SL}\ +\ \mathrm{RL})\end{aligned}$$

$$\begin{aligned} & \text{Seedling vigour index II}\\ & = \ \mathrm{Germination}\ \%\ \text{x Seedling Dry Weight}\ (\mathrm{SLDW}\ =\ \mathrm{SDW}\ +\ \mathrm{RDW})\end{aligned}$$

### Phenotypic data analysis

Descriptive statistics, which included range, mean, standard deviation (SD) and coefficient of variation (CV) were calculated using Microsoft Excel. Analysis of variance (ANOVA) for each trait was performed under a completely randomized design using the ‘variability’ package in R version 4.4.1 [26]. From the ANOVA, variance components were used to estimate the broad-sense heritability (h^2^_bs_) of each trait. The model of ANOVA used was

Y_ij_ = µ + g_i_ + e_ij_.

Where, Y_ij_ = Observation of the i^th^ genotype and j^th^ replication.

µ = overall mean.

g_i_ = genotypic effect.

e_ij_ = residual error.

Genotypic variance (V_g_) was estimated as (Ms_g_-Ms_e_)/r, where,

Ms_g_ = mean square of genotype.

Ms_e_ = mean square error.

r = no. of replications.

Error variance (V_e_) is the same as mean square error, and phenotypic variance (V_p_) was calculated as, V_p_ = V_g_ + V_e_/r.

The broad-sense heritability was calculated as,

$${{\mathrm{h}}^{2}}_{\mathrm{bs}}\ =\ {\mathrm{V}}_{\mathrm{g}}/{\mathrm{V}}_{\mathrm{p}}$$

The histogram and correlation analysis were done using the ‘metan’ package in R.

### Genotypic data

A genotypic data file containing 404,033 high-quality single-nucleotide polymorphisms (SNPs) was obtained from Raiya et al. [22]. The genotypic file was filtered in TASSEL (version 5.2.94) [27] with the following parameters: minor allele frequency ≤ 0.05 (SNPs having minor allele frequency less than 5% were discarded), maximum missing sites ≤ 30% (SNPs present in less than 70% of the genotypes were discarded), and maximum heterozygosity of 0.05 (More the 5% genotypes having heterozygosity at a particular SNP were discarded). After the filtering process we obtained 22,893 SNPs, which were then further used for GWAS.

### Population structure and linkage disequilibrium analysis

For estimating the population structure of the association panel we employed both ADMIXTURE (version 1.3.0) in R [28]. To estimate population structure using ADMIXTURE we used all the 22,893 SNPs and employed the maximum-likelihood method via the expectation maximization (EM) algorithm. The analysis was run for K values ranging from 1 to 20 and to determine the optimal number of K, a fivefold cross-validation procedure was conducted for each K. The cross-validation error for each K value was plotted using the ‘ggplot2’ package in R. The barplot for the Q matrix was visualised using the ‘pophelper’ (version 2.3.1) package in R [29].

Genome-wide linkage disequilibrium analyses were performed in the association panel to evaluate the resolution of linkage disequilibrium (LD). LD between SNPs was calculated pairwise in TASSEL using r^2^, a sliding window of 50 markers, and a minor allele frequency threshold of 0.05. After filtering the dataset by removing any entries with missing values for distance or r^2^, we applied a nonlinear regression model described by Hill and Weir [30] and implemented by Remington et al. [31]. An estimate was obtained from the fitted model for the recombination parameter (*p*) which was used to adjust the r^2^ values relative to genomic distance. These adjusted r^2^ values were then plotted against the physical distance and the half-decay distance was determined.

### Genome-wide association study and identification of candidate genes

In the present study, GWAS was conducted using two models FarmCPU (Li et al., 2016) and BLINK [32] using the ‘GAPIT3’ package (version 3.4.0) [33, 34]. We used the phenotypic data of 230 genotypes and genotypic data containing 22,893 SNPs. Manhattan and Quantile–Quantile (QQ) plots were generated using the GAPIT3 package and significant marker-trait associations were selected based on Bonferroni correction at the significance level of 5%. The threshold was calculated as –log_10_ (0.05/22893) which amounted to a –log_10_ (p) value of 5.66. MTAs exceeding the threshold value in either of the models were considered significant and used for further analysis. Since the Bonferroni correction was found to be overly conservative and we were unable to obtain significant MTAs for some traits, we chose to screen out a set of 11 SNPs associated with Germination percentage, SL, RL, TSL and SVI using a compromised threshold of –log_10_ (1/22893) [35] with a –log_10_ (p) value of 4.36. [36]. GAPIT calculated the MTAs’ percentage of total phenotypic variation (PVE) by dividing their corresponding variance by the total variance, which is the sum of the related markers' variance and residual variance.

We searched for potential candidate genes within a ± 500 kb window flanking each significant MTA, based on our LD decay estimation. JBrowser at Cicerseq (https://cegresources.icrisat.org/cicerseq/) was used to search for putative genes associated with significant MTAs. The BLASTX against the NCBI non-redundant protein database was used to annotate the protein sequences. The candidate genes’ molecular functions were analysed using the UniProt (https://www.uniprot.org/) and QuickGO (https://www.ebi.ac.uk/QuickGO/) databases. A thorough study of literature was also done to associate and understand the function of the candidate genes in relation to ESV.

### Identification of favorable alleles

Allelic variation was investigated by classifying the population's genotypes according to their alleles present at the significant MTAs. Differences in phenotypic means across allelic classes were assessed using the paired t-test with Bonferroni correction to test their significance. To display the variations in trait values among allelic groups, boxplots with significant annotations were used. The statistical analysis and generation of boxplots were done using ‘rstatix’, ‘ggpubr’, and ‘ggplot2’ packages in R.

## Results

### Genetic variability of ESV traits

**Fig. 1:**
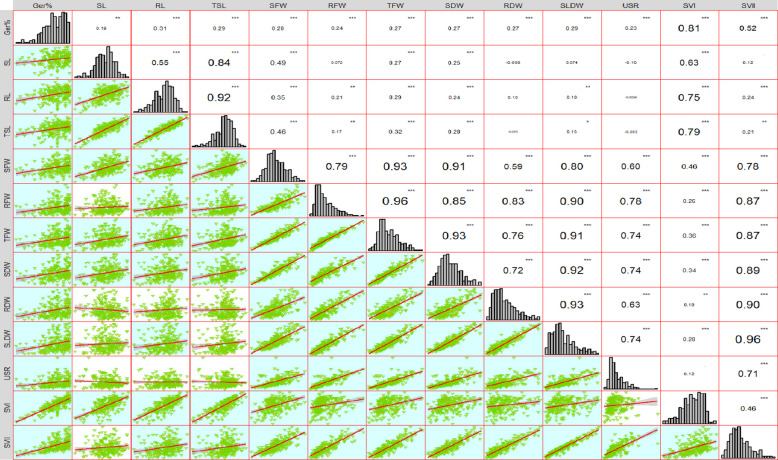
Frequency distribution and correlation coefficients of all the traits in association panel

ANOVA analysis revealed that all 13 traits we used in our current study to map early seedling vigour showed considerable variation (*P <* 0.001) (Table 1). The range of variation, mean, SD, CV, and h^2^_bs_ of the 230 genotypes are presented in Table 2. The Germination %, RL, TSL and SVI were the only traits with a negatively skewed distribution; whereas the rest of the traits showed weak to moderately positively skewed distributions, with the histograms of SL and SFW near normal distribution. Biomass-related traits (FW, DW) displayed a high SD suggesting high genetic diversity of the association panel. Most of the traits displayed high heritability indicating the significance of genetic effects on these traits. Pearson’s correlation coefficient was calculated across all ESV traits. Most of the traits showed high positive correlation (*P <* 0.01) with some exceptions. Non-significant correlation was observed between the following traits- SL with RFW, SLDW, SVII; USR with SVI and negative correlations were observed for USR with SL, RL, TSL and SL with RDW. These results indicate that all the traits selected for ESV evaluation can be used for GWAS studies and they have been shown in Fig. 1.

**Table 1:** Descriptive statistics for ESV traits in the association panel

Traits	Range	Mean	SD	CV %	Broad sense heritability
Germination %	44–100	87.04	10.52	12.09	0.67
Shoot Length(cm)	4.46–16.74	10.41	1.719	16.51	0.71
Root Length(cm)	12.48–26.38	21.08	2.33	11.06	0.74
Total Seedling Length(cm)	19–40.08	31.49	3.57	11.35	0.83
Shoot Fresh Weight(mg)	110.4–551.2	305.98	77.80	25.42	0.80
Root Fresh Weight(mg)	120.04–706.2	283.93	99.53	35.06	0.92
Total Fresh Weight(mg)	260.18–1166.8	589.91	167.82	28.45	0.91
Shoot Dry Weight(mg)	9.6–44.6	23.30	6.48	27.82	0.86
Root Dry Weight(mg)	2–39	12.37	7.068	57.13	0.90
Seedling Dry Weight(mg)	14.6–78.4	35.67	12.59	35.28	0.91
Use of Seed Reserves(mg/seed)	26.24–248.8	78.70	29.91	38.01	0.88
Seedling Vigour Index I	1216–3838	2752.96	507.39	18.43	0.75
Seedling Vigour Index II	776.16–7840	3144.27	1265.03	40.23	0.89

**Table 2:** Analysis of Variance (ANOVA) for 230 chickpea genotypes for 13 ESV traits

S. no	Trait	Sum sq	Mean Sq	*F* value	*P* value
1	Germination%	50,715	221.463	5.0309	< 2 × 10^–16^***
2	SL	1353.94	5.9124	5.8457	< 2 × 10^–16^***
3	RL	2487.07	10.8605	6.7401	< 2 × 10^–16^***
4	TSL	5846.3	25.5297	10.729	< 2 × 10^–16^***
5	SFW	2,772,051	12,105	8.8503	< 2 × 10^–16^***
6	RFW	4,537,449	19,814.2	26.9292	< 2 × 10^–16^***
7	TFW	12,900,342	56,333	20.5399	< 2 × 10^–16^***
8	SDW	19,246.5	84.046	13.4098	< 2 × 10^–16^***
9	RDW	22,882.8	99.925	18.6669	< 2 × 10^–16^***
10	SLDW	72,545	316.79	22.3912	< 2 × 10^–16^***
11	USR	409,846	1789.72	16.2948	< 2 × 10^–16^***
12	SVI	117,911,736	514,898	6.9878	< 2 × 10^–16^***
13	SVII	732,939,356	3,200,609	17.582	< 2 × 10^–16^***

### SNP distribution, population genetic structure, and linkage disequilibrium

**Fig. 2:**
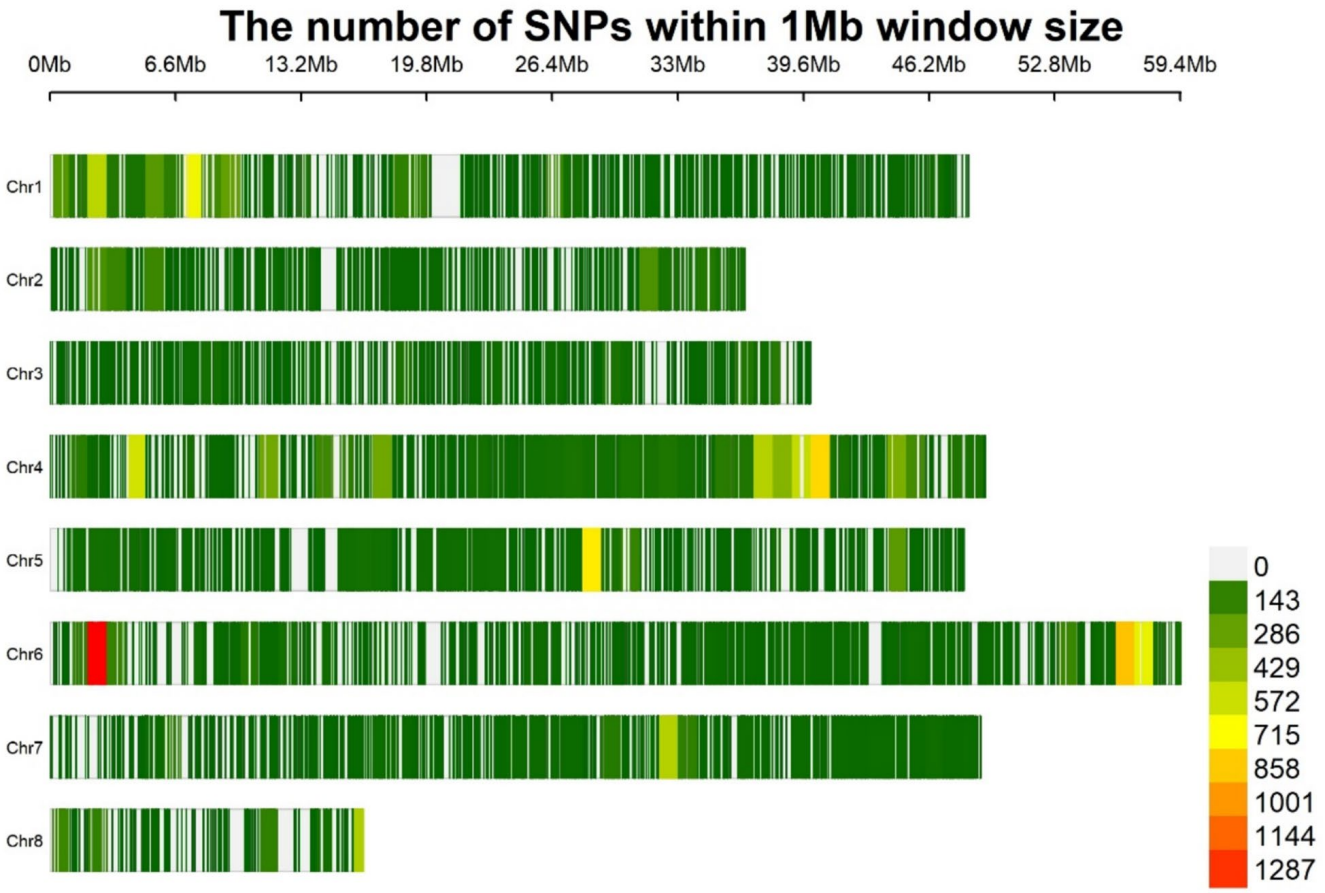
SNP density plot showing the distribution of SNPs across all chromosomes

For the GWAS analysis, a pruned dataset of 22,893 SNPS was used after filtering. The number of SNPs per chromosome ranged from 1118 to 6017, with an average of 2862 SNPs per chromosome. The highest number of SNPs was observed on chromosome 4 (6017) and 6 (4834), while the lowest number of SNPs was in chromosome 3 (1176) and 8 (1118). The distribution of the SNPs along the eight chromosomes is shown as a marker density plot in Fig. 2.

**Fig. 3:**
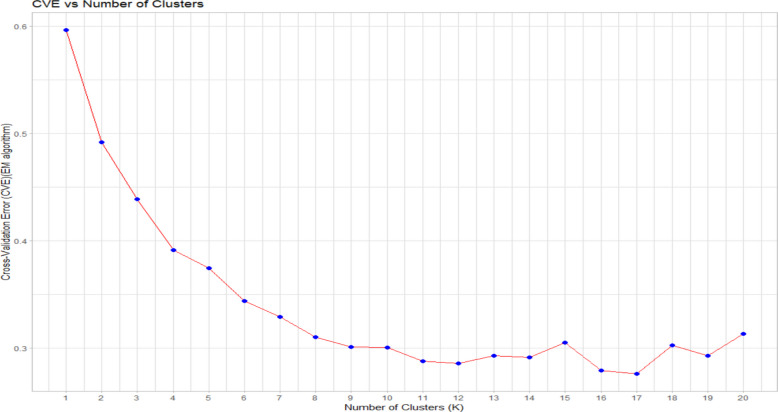
Cross validation error for K = 1–20 in ADMIXTURE analysis

**Fig. 4:**
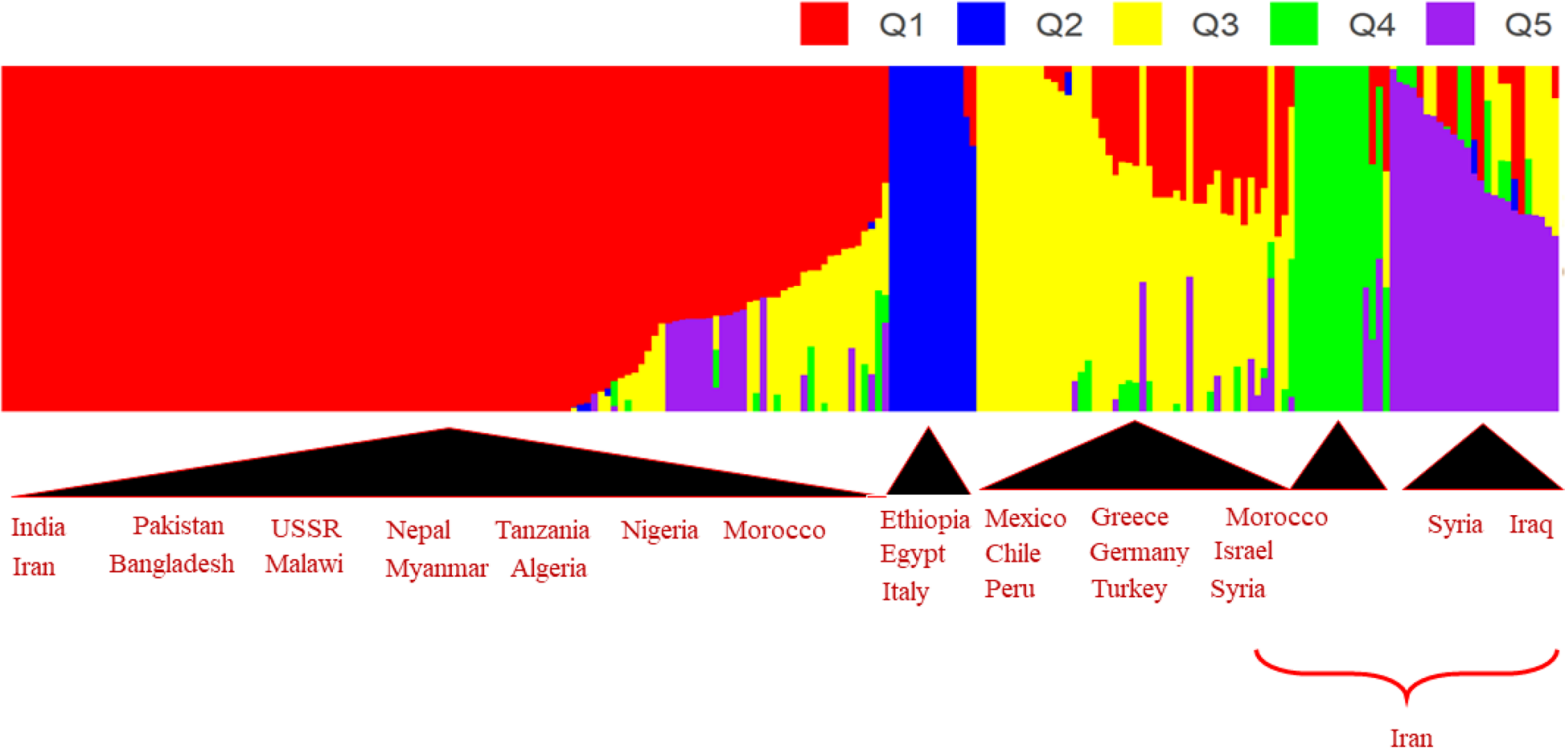
Barplot of ancestry proportions for K = 5 from ADMIXTURE analysis

The cross-validation error (CVE) of population structure analysis in ADMIXTURE indicated that the CVE dropped significantly from 0.597 to 0.344 with increasing K from 1 to 6. The lowest CVE estimates were recorded at K values of 12, 16, and 17 (Fig. 3). Using the Q matrix K values of 3–6 were screened for population structure analysis. When using the Q matrix for K = 5 we observed that it was able to cluster the genotypes almost perfectly according to their geographical origin with some minor exceptions. The first subpopulation was composed mainly of genotypes from three regions. One originated from the Asian countries, India, Pakistan, Bangladesh, Myanmar, Russia and Afghanistan, the other from the North-Western African countries, Algeria, Morocco and Nigeria. The third region was made up of the South-Eastern African countries of Tanzania and Malawi. Ancestry proportion of Indian-origin genotypes were predominantly > 0.7 in the first cluster. Genotypes from Ethiopia made the second subpopulation, with one genotype from Egypt and Italy also included indicating their possible Ethiopian origin. Similar to the first subpopulation, subpopulation 3 also had genotypes from three different regions. It included lines from European countries Greece and Germany, American countries USA, Peru, Chile and Mexico and Western Asian countries Israel, Turkey and Syria. The fourth subpopulation had Iran-origin genotypes and one line from Italy. Genotypes originating from Iran were present in four out of the five subpopulations owing to their highly mixed ancestry proportions. Also, genotypes from Afghanistan, Syria and Turkey were found in two out of the five subpopulations and were having mixed ancestry proportions. The barplot of the ancestry proportions for K = 5 is shown in Fig. 4. We couldn’t use the ancestry proportion to correctly group the genotypes by their geographical origin when looking at the Q-matrix values for K values of 3, 4, 6, 12 and 17. The population structure of our association panel was both complex and cryptic, with most genotypes having mixed ancestral proportions. Therefore, we chose to partition our panel into 5 population clusters for the GWAS analysis.

**Fig. 5:**
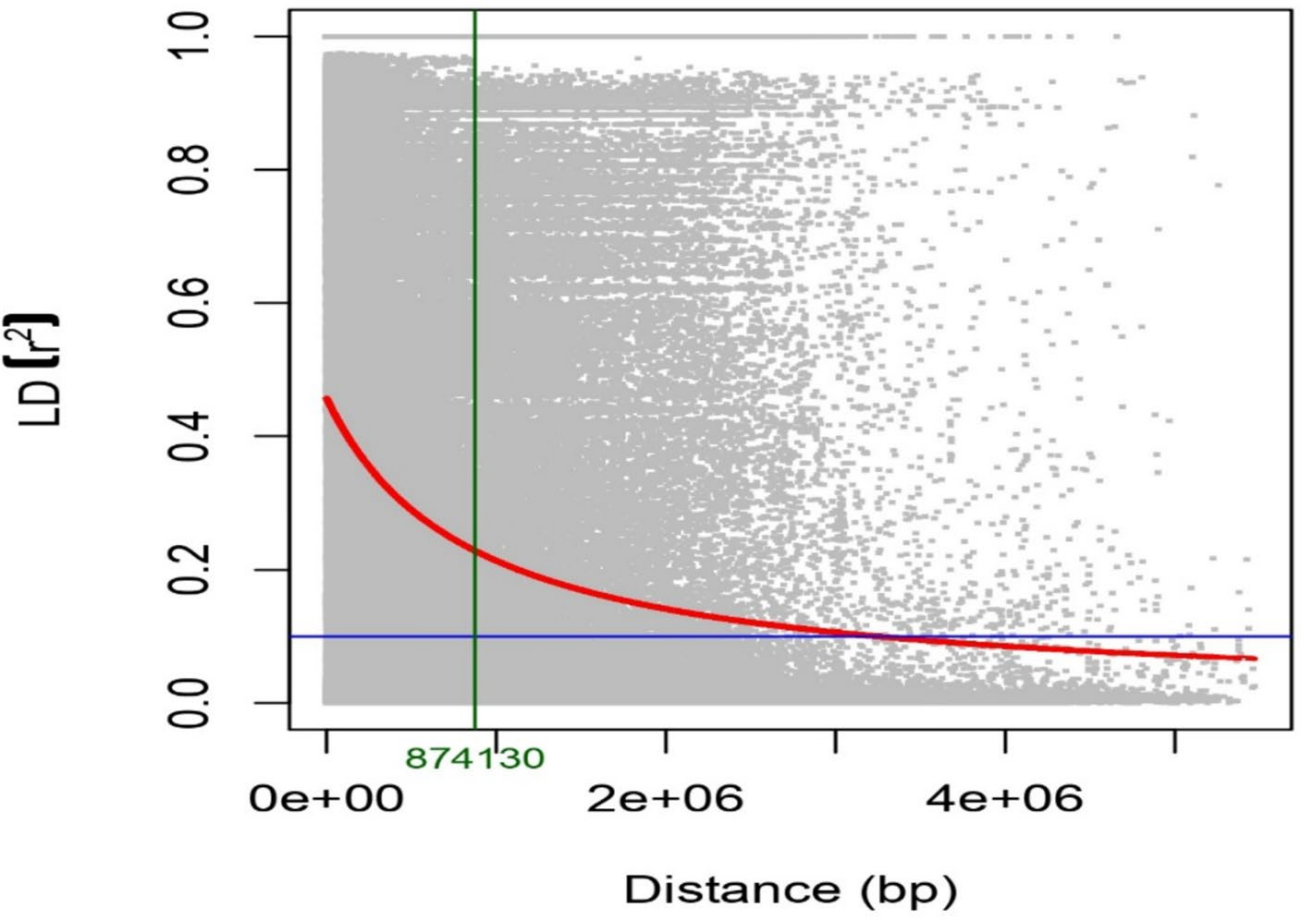
Whole-genome linkage disequilibrium in the association panel

Linkage disequilibrium (LD) decay was used to estimate the extent of genomic relationship among the SNP markers used in the study. The average r^2^ decreased to < 0.2 at about 874 kb, regarded as the extent of LD decay of the population (Fig. 5).

### Identification of significant MTAs linked to ESV traits

**Fig. 6:**
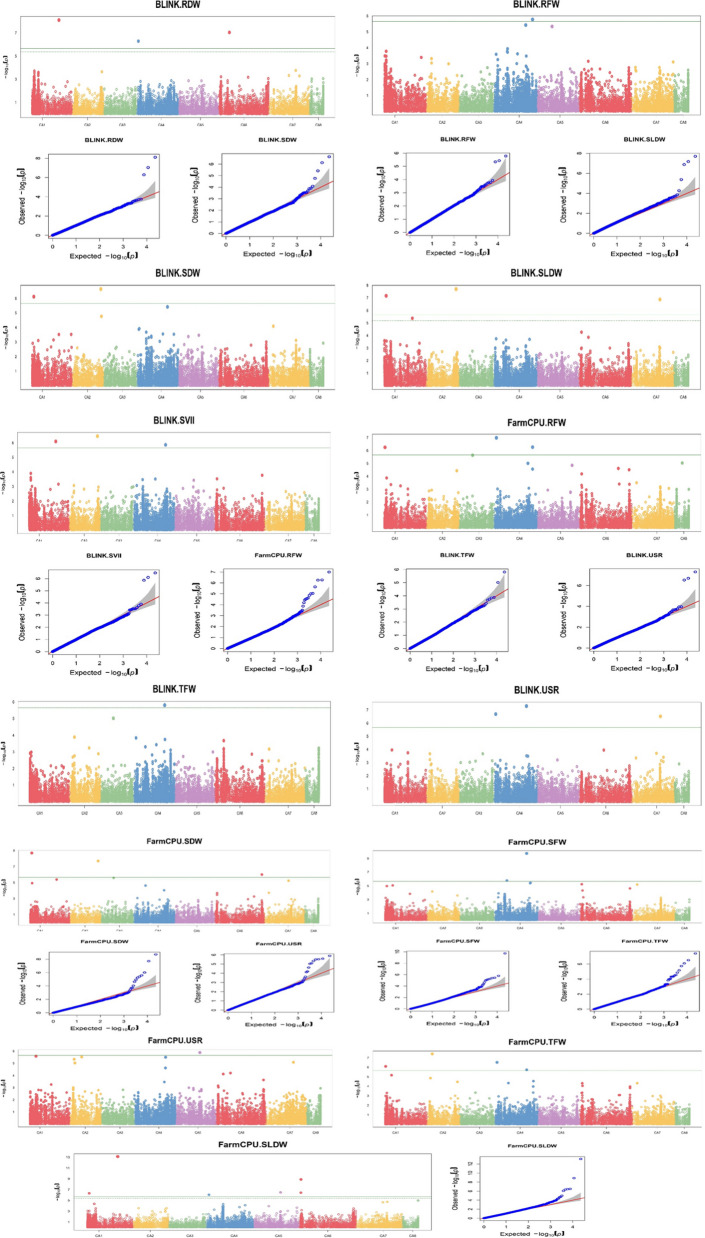
Manhattan and QQ plots of significant associations for ESV traits

**Fig. 7:**
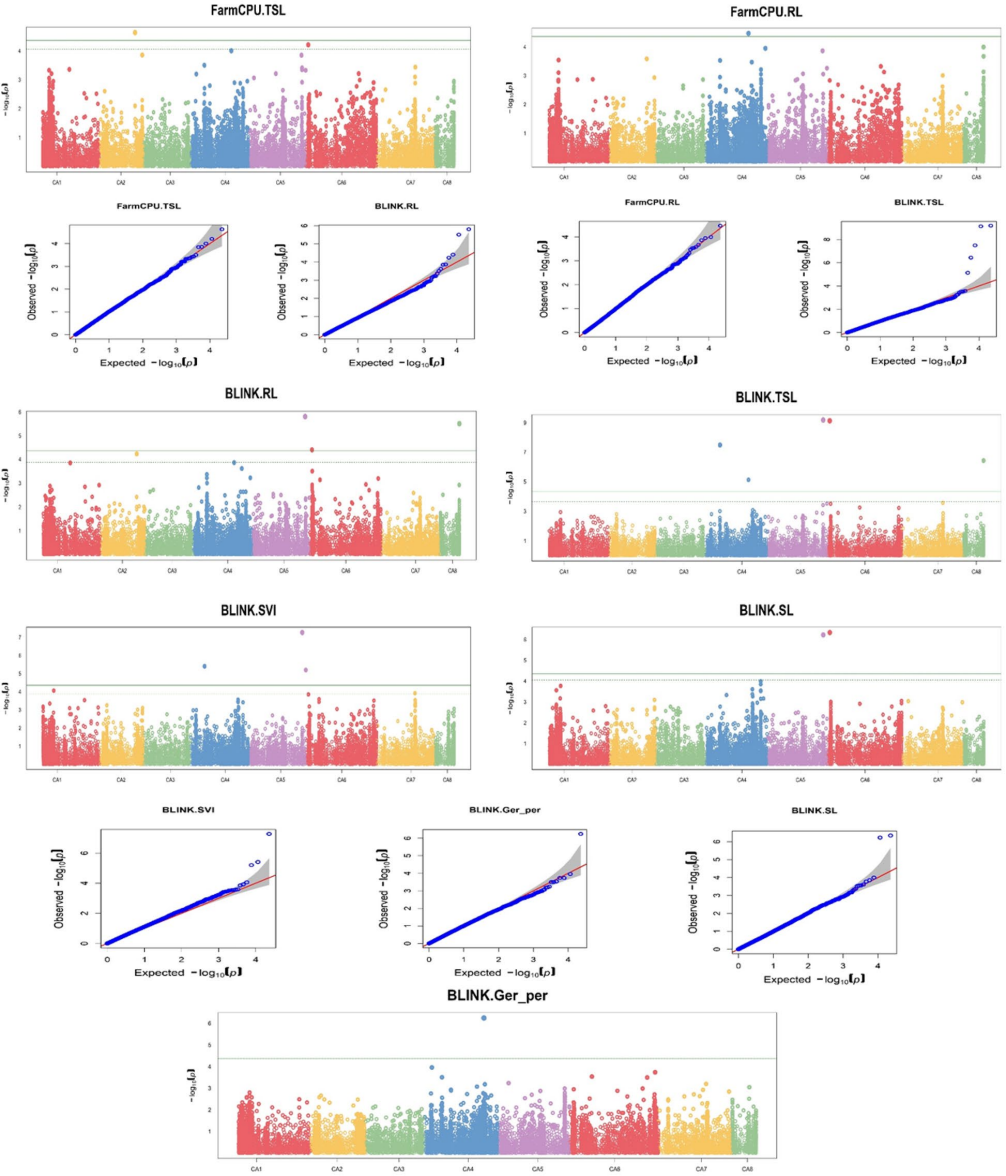
Manhattan and QQ plots of associations obtained after lowering the Bonferroni threshold

GWAS analyses were conducted on all 13 traits using two models in GAPIT/R. Genome-wide association analysis identified 23 MTAs linked with 8 of the traits using a Bonferroni threshold at a –log (p) value of 5.66 to identify statistically significant MTAs (Table 3). BLINK model identified 11 significant MTAs associated with RFW, TFW, SDW, RDW, SLDW, USR and SVII. Utilising the FarmCPU model, we identified 15 significant MTAs associated with SFW, RFW, TFW, SDW, SLDW and USR traits. Most significant MTAs were detected for SLDW (9), followed by SDW (5) and TFW (5) (Fig. 6). Most of the MTAs were detected in LG 4 (10), followed by LG 1 and LG 6, both of which had 6 each. No significant MTAs were detected in LG 3 and LG 8. From our analysis we identified some MTAs having high PVE for their particular trait. One MTA Ca1_32048812 showed 47.9% and 62.1% PVE for RDW and SVII respectively. Other MTAs also showed a high percentage of PVE, for instance, Ca2_33654712 (35.2% PVE for SDW) and Ca2_5629077 (33.93% PVE for trait TFW). The said MTAs are listed in detail in Table 2. In our study, we were unable to find significant associations for the traits Germination%, SL, RL and SVI with the initially determined Bonferroni threshold. As Bonferroni correction is found to be overly conservative [73] we decided to look for MTAs for the remaining trait by lowering the Bonferroni threshold to a –log (p) value of 4.36. We identified 11 MTAs linked to Germination% (1), SL (2), RL (4), TSL (6) and SVI (3) (Fig. 7). Among them Ca4_34378091 was detected in both the models for RL and TSL. Ca5_44805420 and Ca6_1986291 were detected for multiple traits. The list of Bonferroni-corrected MTAs is presented in Table 4.

**Table 3:** List of significant MTAs for ESV traits

S. no	SNP	Chromosome	Position	P value	-log_10_(p) value	MAF	Model	Traits
1	Ca1_2192664	CA1	2,192,664	7.62E-07	6.12	0.16	BLINK	SDW
				2.01E-09	8.70		FarmCPU	SDW
				6.75E-08	7.17		BLINK	SLDW
2	Ca1_2192687	CA1	2,192,687	4.78E-07	6.32	0.16	FarmCPU	SLDW
3	Ca1_32048812	CA1	32,048,812	7.80E-09	8.11	0.12	BLINK	RDW
				7.99E-07	6.10		BLINK	SVII
				7.61E-14	13.12		FarmCPU	SLDW
4	Ca1_815429	CA1	815,429	5.64E-07	6.25	0.19	FarmCPU	RFW
				8.04E-07	6.09		FarmCPU	TFW
5	Ca2_33654712	CA2	33,654,712	1.99E-08	7.70	0.13	BLINK	SLDW
				3.43E-07	6.46		BLINK	SVII
				1.99E-08	7.70		FarmCPU	SDW
6	Ca2_5629077	CA2	5,629,077	3.91E-08	7.41	0.16	FarmCPU	TFW
7	Ca4_14134993	CA4	14,134,993	1.64E-06	5.78	0.21	FarmCPU	SFW
8	Ca4_1860828	CA4	1,860,828	5.15E-07	6.29	0.21	BLINK	RDW
9	Ca4_2130281	CA4	2,130,281	2.03E-07	6.69	0.19	BLINK	USR
10	Ca4_2873542	CA4	2,873,542	1.03E-07	6.99	0.15	FarmCPU	RFW
				3.06E-07	6.51		FarmCPU	TFW
				8.96E-07	6.05		FarmCPU	SLDW
11	Ca4_36632427	CA4	36,632,427	1.86E-10	9.73	0.25	FarmCPU	SFW
				1.85E-06	5.73		FarmCPU	TFW
12	Ca4_37104463	CA4	37,104,463	4.96E-08	7.30	0.26	BLINK	USR
13	Ca4_37245599	CA4	37,245,599	1.56E-06	5.81	0.12	BLINK	TFW
14	Ca4_38647446	CA4	38,647,446	1.37E-06	5.86	0.28	BLINK	SVII
15	Ca4_44080221	CA4	44,080,221	5.45E-07	6.26	0.10	FarmCPU	RFW
16	Ca4_44380810	CA4	44,380,810	1.68E-06	5.77	0.15	BLINK	RFW
17	Ca5_28695937	CA5	28,695,937	3.28E-07	6.48	0.26	FarmCPU	SLDW
18	Ca5_28931405	CA5	28,931,405	1.29E-06	5.89	0.22	FarmCPU	USR
19	Ca6_12971377	CA6	12,971,377	9.08E-08	7.04	0.23	BLINK	RDW
20	Ca6_1942884	CA6	1,942,884	3.67E-07	6.43	0.12	FarmCPU	SLDW
21	Ca6_2047853	CA6	2,047,853	1.31E-09	8.88	0.13	FarmCPU	SLDW
22	Ca6_56851553	CA6	56,851,553	9.85E-07	6.01	0.15	FarmCPU	SDW
23	Ca7_33017177	CA7	33,017,177	1.33E-07	6.87	0.18	BLINK	SLDW
				3.00E-07	6.52		BLINK	USR

**Table 4:** List of Bonferroni-corrected MTAs

S. no	SNP	Chromosome	Position	P value	-log_10_(p) value	MAF	Model	Traits
1	Ca2_29742379	CA2	29,742,379	2.35E-05	4.63	0.14	FarmCPU	TSL
2	Ca4_11666238	CA4	11,666,238	3.93E-06	5.41	0.21	BLINK	SVI
3	Ca4_11685517	CA4	11,685,517	3.21E-08	7.49	0.18	BLINK	TSL
4	Ca4_34378091	CA4	34,378,091	7.31E-06	5.14	0.15	BLINK	TSL
				3.40E-05	4.47		FarmCPU	RL
5	Ca4_39744712	CA4	39,744,712	5.71E-07	6.24	0.17	BLINK	Ger_per
6	Ca5_44322104	CA5	44,322,104	1.57E-06	5.80	0.11	BLINK	RL
7	Ca5_44805420	CA5	44,805,420	5.97E-07	6.22	0.22	BLINK	SL
				5.39E-08	7.27		BLINK	SVI
				6.52E-10	9.19		BLINK	TSL
8	Ca5_47801558	CA5	47,801,558	6.29E-06	5.20	0.16	BLINK	SVI
9	Ca6_1986291	CA6	1,986,291	3.94E-05	4.40	0.16	BLINK	RL
				4.58E-07	6.34			SL
				7.36E-10	9.13			TSL
10	Ca8_16353912	CA8	16,353,912	3.12E-06	5.51	0.09	BLINK	RL
11	Ca8_16380639	CA8	16,380,639	3.65E-07	6.44	0.12	BLINK	TSL

### Identification of candidate genes and their allelic variations associated with ESV traits

Our search for candidate genes among important SNPs mainly focused on SNPs that had been detected by more than one GWAS model, SNP alleles that were significantly different in phenotypic data, and SNPs that explained a larger fraction of the phenotypic variance. The most significant SNPs were intergenic, based on their physical location in a reference genome; four SNPs were located inside genes, but all were located within introns. After finding relevant genes in the reference genome and annotating them with UniProt and QuickGo, we investigated further literature to learn more about their roles and potential relationships to ESV traits. We initially identified 27 possible candidate genes linked to significant MTAs (Table 5). It includes genes responsible for preliminary shoot and root growth and hormone signalling. Bonferroni corrected MTAs also showed a close association with important genes related to seedling growth and vigour in plants. Additionally we identified 9 candidate genes linked to the MTAs. 4 MTAs were present in the intronic region of genes and others were present in the intergenic region. Some more genes involved in cell division, maintenance of meristems and auxin signalling have also been identified which are imperative for early seedling vigour and the whole list is presented in Table 6.

**Table 5:** List of genes associated with MTAs

SNP	Traits	Gene ID	Protein name
Ca4_37104463	USR	Ca_15155	TREHALOSE-6-PHOSPHATE SYNTHASE DOMAIN PROTEIN
Ca4_44380810	RFW	Ca_09072	ABERRANT ROOT FORMATION PROTEIN 4
Ca4_37245599	TFW	Ca_15151	B-TYPE RESPONSE REGULATOR
		Ca_14799	PROTEIN ROOT INITIATION DEFECTIVE 3
Ca1_2192664	SDW, SLDW	Ca_00275	SERINE/THREONINE-PROTEIN PHOSPHATASE 7 LONG FORM HOMOLOG
Ca2_33654712	SDW, SLDW, SVII	Ca_10175	PROTEIN LATERAL ROOT PRIMORDIUM 1
Ca1_32048812	RDW, SVII, SLDW	Ca_28114	OBERON-LIKE PROTEIN
Ca4_1860828	RDW	Ca_07788	AUXIN-RESPONSIVE AUX/IAA FAMILY PROTEIN
Ca6_12971377	RDW	Ca_05069	PROLYL 3-HYDROXYLASE 1
Ca7_33017177	SLDW, USR	Ca_10012	GROWTH-REGULATING FACTOR 5-LIKE ISOFORM X1
		Ca_10009	GERMIN FAMILY 3 PROTEIN
Ca4_2130281	USR	Ca_07833	RHO GTPASE
Ca4_38647446	SVII	Ca_13101	GRAS FAMILY TRANSCRIPTION FACTOR
		Ca_13102	SCARECROW-LIKE PROTEIN 1
Ca4_14134993	SFW	Ca_04624	FANTASTIC FOUR-LIKE PROTEIN (FAF 3)
		Ca_04629	HOMEOBOX-LEUCINE ZIPPER PROTEIN MERISTEM L1-LIKE
Ca4_36632427	SFW, TFW	Ca_14811	bZIP TRANSCRIPTION FACTOR (G-BOX-BINDING FACTOR 1-LIKE)
Ca1_815429	RFW, TFW	Ca_00099	AUXIN-INDUCED 5NG4-LIKE PROTEIN
Ca4_2873542	RFW, TFW, SLDW	Ca_12081	ETHYLENE-RESPONSIVE TRANSCRIPTION FACTOR 1B
Ca4_44080221	RFW	Ca_09081	CHD3-TYPE CHROMATIN-REMODELING FACTOR PICKLE ISOFORM X2
Ca2_5629077	TFW	Ca_19701	WUSCHEL-RELATED HOMEOBOX 11
Ca6_56851553	SDW	Ca_17438	COBRA-LIKE PROTEIN 4
Ca1_2192687	SLDW	Ca_00275	SERINE/THREONINE-PROTEIN PHOSPHATASE 7 LONG FORM HOMOLOG
Ca5_28695937	SLDW	Ca_13379	SNF2 DOMAIN-CONTAINING PROTEIN CLASSY 2-LIKE
Ca6_1942884	SLDW	Ca_10316	WUSCHEL-RELATED HOMEOBOX 7-LIKE
Ca6_2047853	SLDW	Ca_10335	ABC TRANSPORTER G FAMILY MEMBER 18-LIKE
Ca5_28931405	USR	Ca_13358	PEPTIDE/NITRATE TRANSPORTER (NRT1/PTR FAMILY 5.1)
Ca2_29742379	TSL	Ca_16131	TRANSCRIPTION FACTOR MYB3-LIKE
Ca4_11666238	SVI	Ca_04390	PROTEIN ROOT PRIMORDIUM DEFECTIVE 1/PROTEIN WHAT'S THIS FACTOR 1 HOMOLOG, CHLOROPLASTIC
Ca4_11685517	TSL	Ca_04390	PROTEIN ROOT PRIMORDIUM DEFECTIVE 1/PROTEIN WHAT'S THIS FACTOR 1 HOMOLOG, CHLOROPLASTIC
Ca4_34378091	RL, TSL	Ca_20371	PROTEIN SMAX1-LIKE 3-LIKE
Ca4_39744712	Ger_per	Ca_14916	AQUAPORIN TIP4-1-LIKE
Ca5_44322104	RL	Ca_03879	PROTEIN KRTCAP2 HOMOLOG
Ca5_44805420	SL, TSL, SVI	Ca_03924	BIDIRECTIONAL SUGAR TRANSPORTER SWEET1-LIKE
Ca5_47801558	SVI	Ca_04245	AUXIN-RESPONSIVE PROTEIN SAUR32-LIKE
Ca6_1986291	SL, RL, TSL	Ca_10366	MOTHER OF FT AND TFL1-LIKE PROTEIN
Ca8_16353912	RL	Ca_15558	CYCLOARTENOL SYNTHASE
Ca8_16380639	TSL	Ca_15558	CYCLOARTENOL SYNTHASE

**Table 6:** List of putative candidate genes for ESV and their biological functions

MTA	Gene ID	Gene name	Biological functions	References
Ca4_44380810	LOC101496540	aberrant root formation protein 4	Involved in lateral root formation	[37, 38]
Ca4_37245599	LOC101510474	B-type response regulator/two-component response regulator ORR21	Contributes to cytokinin signal transduction pathway required for proper root development	[39]
	LOC101500401	protein ROOT INITIATION DEFECTIVE 3	Role in meristem maintenance, leaf morphogenesis, and root morphogenesis	[40]
Ca1_2192664, Ca1_2192687	LOC101498770	serine/threonine-protein phosphatase 7 long form homolog/protein MAIN-LIKE 1-like	Required for the organization of the root apical meristem (RAM) and the shoot apical meristem (SAM)	[76]
Ca2_33654712	LOC101498687	protein LATERAL ROOT PRIMORDIUM 1	Involved in early lateral root formation	[41]
Ca1_32048812	LOC101506156	OBERON-like protein	Crucial for the maintenance and/or establishment of both the shoot and root meristems	[42, 43]
Ca4_1860828	LOC101514996	auxin-responsive AUX/IAA family protein/auxin-induced protein 22D-like	Involved in primary and lateral root formation	[44]
Ca4_38647446	LOC101496871	GRAS family transcription factor	Similar to scarecrow-like protein	
	LOC101496871	scarecrow-like protein 1	SCR is a key regulator of ground tissue patterning in the root by regulating asymmetric cell division and endodermis differentiation	[45]
Ca4_14134993	LOC101503509	fantastic four-like protein (FAF 3)	Regulate the size of the shoot meristem by modulating the CLV3-WUS feedback loop	[46]
	LOC101508445	homeobox-leucine zipper protein MERISTEM L1-like	Probable transcription factor involved in cell specification and pattern formation during embryogenesis	[47]
Ca4_44080221	LOC101500650	CHD3-type chromatin-remodeling factor PICKLE isoform X2	Required for the activation of root stem cell and meristem marker genes	[48, 49]
	LOC101500109	Two-component response regulator ARR12	ARR12 is involved in the AHK-dependent signaling pathway that modulates the differentiation of root vascular tissues	[50, 51]
Ca2_5629077	LOC101496040	WUSCHEL-related homeobox 11	Role in regulating the development of adventitious roots	[52]
Ca6_1942884	LOC101506764	WUSCHEL-related homeobox 7-like	Role in lateral root development	[52]
Ca2_29742379	LOC101490995	Transcription factor MYB3-like	Has possible role in germination and seedling establishment	[53]
Ca4_11666238, Ca4_11685517	LOC101497060	protein ROOT PRIMORDIUM DEFECTIVE 1/protein WHAT'S THIS FACTOR 1 homolog, chloroplastic	Involved in pre-arranging the maintenance of the active cell proliferation during root primordium development	[54]
Ca4_34378091	LOC101508230	protein SMAX1-LIKE 3-like	SMAX1 integrates multiple growth hormone signals into optimizing seedling establishment	[55]
Ca4_39744712	LOC101505122	Aquaporin TIP4-1-like	Possible candidate for driving the rapid cellular expansion associated with post-germination seedling growth	[56]
Ca6_1986291	LOC101504081	mother of FT and TFL1-like protein	Regulates seed germination and post-germination growth through integrating GA and ABA signalling in *Arabidopsis*	[57]

**Fig. 8:**
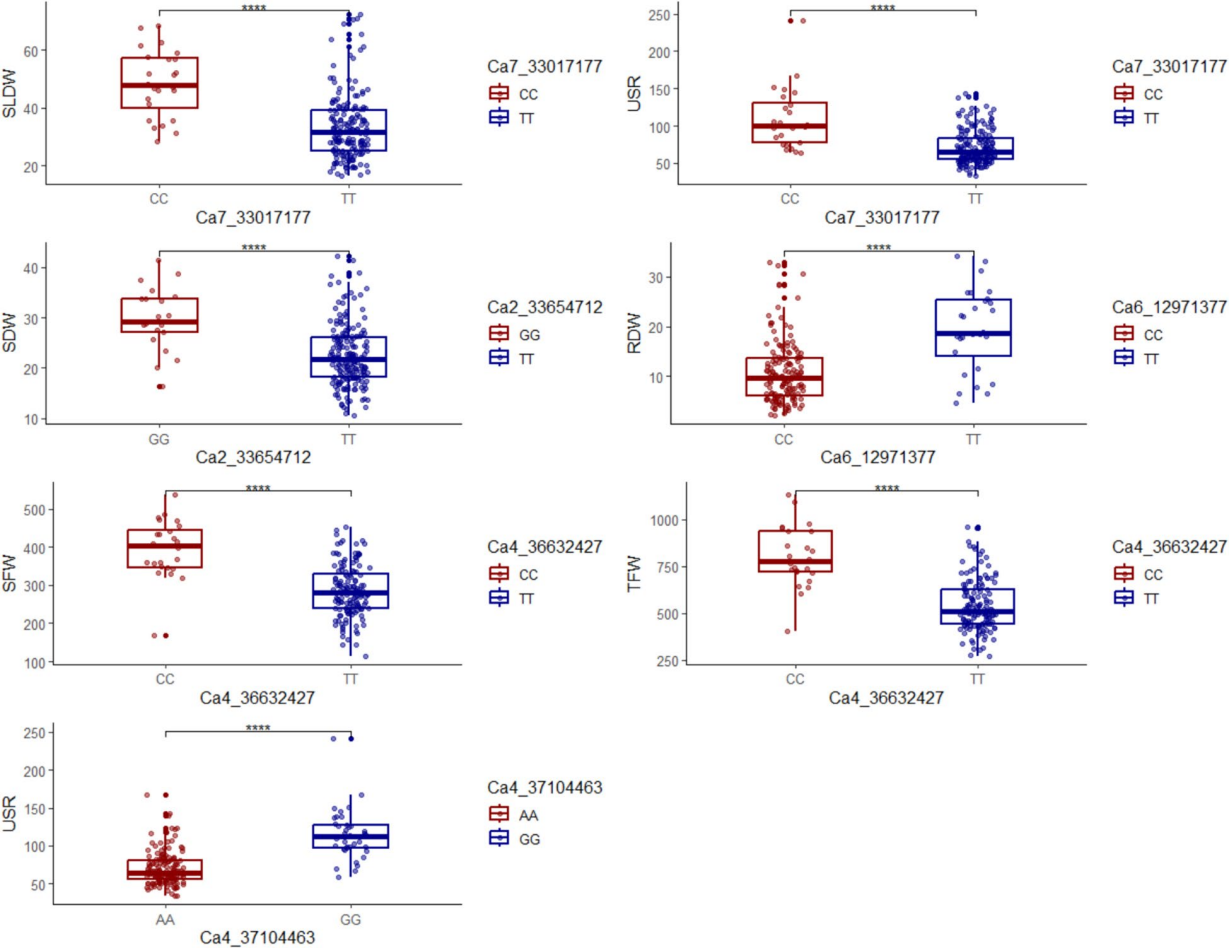
Boxplots of allelic effects of significant MTAs

It is important to identify the superior alleles linked to important traits. Their identification may facilitate the improvement of crops through marker-assisted selection (MAS) and genomic selection (GS). In our study we have identified superior alleles for five MTAs. The genotypes in the association panel can be categorised based on their particular allele on that MTA. The boxplots indicate the effect of one allele over the other with respect to their phenotypic data and they are shown in Fig. 8.

## Discussion

Self-sufficiency of food staple crops is one of the main drivers of global food security as it stabilises local food availability, mitigates against supply-chain stocks and reduces reliance on food imports. It is critical to a country’s attainment of food security and sovereignty. India is one of the world’s largest producers of chickpea, contributing to the production of about 75 per cent of the world's volatile chickpea output, but and ranks as one of the largest chickpea importers with chickpea imports totalling about $172 million [58]. Chickpea being an important crop, increasing the area under cultivation of chickpea, the importance of the shift from fallow to chickpea versus rice and favouring the rice-chickpea cropping pattern becomes important for increasing the productivity, promoting sustainable intensification of agriculture and achieving self-sufficiency. However, rice-fallow has its own limitations such as—low water holding of the soil, depletion of soil moisture, heavy weed population and poor soil structure which results in poor crop stand. For subsequent crop growth to be successful, good crop establishment is essential. Early vigour of seedlings is a trait of high-quality seeds that refers to the quick, consistent germination and establishment of robust seedlings under varied environmental circumstances [5]. Varieties with ESV help suppress weed growth, decrease soil evaporation, speed up root access to soil water and nitrogen and provide quick ground cover at the vegetative stage [59]. Therefore, it is important to study early seedling vigour in chickpea to develop genotypes suitable for the rice-chickpea cropping system. This present study is the first in nature to report the genetics of ESV in chickpea. ESV is a highly complex trait, as it is not determined by a single characteristic but rather encompasses a broad spectrum of morphological, physiological and biochemical attributes. In most studies to assess seedling vigour, morphological traits are commonly measured like mean germination time, seedling shoot and root length, fresh weight, early growth rate, seedling leaf area, germination index, seedling vigour index I & II etc. These traits have been used for mapping in rice, barley, finger millet and soybean [14, 15, 60, 61]. In our study we had taken all morphological traits to assess the ESV of the association panel and there were phenotypically significant variations for the traits. Analysis of variance revealed that variances due to genotype were significant (*P <* 0.001) for all of the traits. The fact that nearly every trait has a strong positive correlation with seedling vigour I and II indicates that it may be feasible to select for any of them in order to increase seedling vigour in genotypes. We got sufficient variability for biomass-related traits in the association panel. Given that the most prevalent indicator of seedling vigour is rapid dry matter generation during early growth, high early vigour may be associated with a high relative growth rate during the early stages of seedling development [62]. All traits showed high heritability ranging from 0.67 for germination percentage to > 0.9 for traits like RFW, TFW and SLDW. Due to their strong genetic regulation and very low environmental influence during this crucial early developmental stage, germination and early growth traits usually show high heritability and similar results have been found in other studies also [60, 74]. These results indicate the significant genetic basis of traits related to germination and early seedling growth.

We have also identified high and low early vigorous lines from our association panel by normalizing the values of both seedling vigour indexes and then adding them. We observed that in general kabuli genotypes had higher biomass accumulation whereas the desi genotypes had higher seedling length and higher germination percentage. Desi lines ICC15567, ICC8318 and kabuli lines ICC20259, ICC20263 and ICC15406 were found to have high early seedling vigour based on our results and visual evaluation. Similarly low early vigorous genotypes were also identified- ICC1164, ICC11279, ICC4814, ICC9702 from desi and ICC12328 from kabuli. These are valuable resources for further investigations aiming at identifying important vigour and growth related genes and/or genomic regions. Identification of contrasting genotypes for early vigour can also help in developing mapping populations for fine mapping and breeding for high early vigorous chickpea cultivars.

The degree of LD throughout the genome determines how many markers are needed for GWAS analysis. The density of markers needed for improved mapping resolution increases with decreasing LD across the genome and vice versa. The distribution and degree of LD throughout the genome determine the major marker-trait associations [63]. In the present study we observed that the LD (r^2^) decay rate was relatively slow (874 kb) across the genome. This indicated that chickpea being a self-pollinating crop has a slower LD decay rate and as most of the population is comprised of landraces, chickpea landraces exhibit slow decay of LD which is in agreement with similar studies [64]. The extent of LD decay in the minicore collection of chickpea, from which our association panel is derived has been observed to be from 100–450 kb [17] to 4032 kb [21]. This implies that variables like the number of genotypes, population structure, SNP marker density, and methodology affect LD decay rate. So we decided to search for candidate genes within ± 500 kb of our associated SNP.

In our study we have determined the association panel to be subdivided into five population clusters based on our population structure analysis. The observed population clusters were SP1 (131 genotypes), SP2 (13 genotypes), SP3 (47 genotypes), SP4 (14 genotypes) and SP5 (25 genotypes). Most of our lines were of Indian origin and most (95%) of them were under SP1. A Q matrix value of ≥ 70 percent was used to set a criterion of non-admixed genotypes. Genotypes who were admixed were categorised if their Q matrix scores were less than 70% [65]. SP1 consisted most of the genotypes and it had around 16% admixed lines. The most proportion of admixed lines were found in SP3 (57%) and SP5 (52%), the lines originating mostly from Afghanistan, Iran and Turkey. As our association panel was derived from the chickpea minicore collection it is expected to be an admixed and diverse population given how it was chosen from the entire core collection to represent maximum diversity [65]. In other studies which included similar chickpea genotypes, population cluster was reported to range from 2 to 4 [17, 19–22, 71]. As mentioned in [71], deciphering the population structure of this panel may sometimes give inconclusive results due to the genotypes having high admixture and mixed ancestral origins, so based on our ADMIXTURE analysis we decided to classify the association panel into five population clusters.

In our study we identified a total of 34 MTAs using two different Bonferroni thresholds for all the traits using FarmCPU and BLINK models. The main objective of our study was to identify putative candidate genes involved directly or indirectly in providing early seedling vigour. We were able to identify a total of 36 putative candidate genes associated with the identified MTAs. Early root development is important for the establishment of plants at the seedling stage, it ensures the plant has better anchorage in the soil, more access to water and nutrients and improves drought resilience. The gene encoding for ABERRANT ROOT FORMATION PROTEIN 4 was found associated with the MTA Ca4_44380810 detected for RFW. This protein, which has been cloned in *Arabidopsis*, encodes a nuclear-localized protein that is necessary for maintaining the capacity of xylem-adjacent pericycle cells to re-differentiate into lateral roots, hence contributing to the production of lateral roots [37, 38]. One candidate gene—LATERAL ROOT PRIMORDIUM 1 was found in the vicinity of the MTA Ca2_33654712. The homologs of this gene in Arabidopsis and maize were found to be involved in the early formation of lateral roots important for robust root growth [41]. The SNP Ca4_37245599 on chromosome 4 for TFW was 2.4 kb away from a potential candidate gene- B-TYPE RESPONSE REGULATOR, whose homolog in *Arabidopsis* has been found to contribute to the cytokinin signal transduction pathway required for proper root development [39]. Another MTA Ca4_44080221 was found to be associated with RFW in our study and we found two genes *Ca_09080* and *Ca_09081* in its vicinity. *Ca_09081* encodes for TWO-COMPONENT RESPONSE REGULATOR ARR12, which is a member of the type-B authentic response regulator (ARR) family. The AHK-dependent signalling system that controls the development of root vascular tissues includes ARR12 [50, 51]. *Ca_09080* translates into CHD3-TYPE CHROMATIN-REMODELING FACTOR PICKLE which has a plethora of biological functions. The PICKLE (PKL) gene has been found in *Arabidopsis* to repress expression of embryonic traits during germination [66]. PKL encodes a CHD3 chromatin remodeler and plays an essential role in root development by controlling root meristem activity [48, 49]. Another important candidate gene was *Ca_07788* encodes for an auxin-responsive AUX/IAA family protein. After BLAST we identified the protein *as Cicer arietinum* AUXIN-INDUCED PROTEIN 22D-LIKE. AUX/IAA proteins are short-lived transcription factors which can act as repressors of early auxin-responsive genes in the absence of auxin [75]. AUX/IAA family genes have been shown to be involved in the primary and lateral root formation [44]. The zinc-finger protein WIP2-like has been found to play a crucial role in lateral root development in chickpea [67]. In this study, no SNPs were identified within that region, though this result may be due to the scope of the SNP set used, or another major reason may be that we are phenotyping at a very early stage. The genes we identified through association with our significant SNPs might be involved in early lateral root formation, just after the development of the primary root. Nevertheless, the function of this novel gene is known, and it may still be considered as a potential candidate for further targeted sequencing and functional validation.

As the shoot apical meristem (SAM) is involved in the development of shoot as well as the early organ initiation, it is possible that it has a dynamic role in the seedling vigour. The developed SAM is the main contributor to the division and differentiation of cells which leads to initiation of the leaf primordia, expansion of the canopy and increased photosynthetic rate which contribute to the rapid rate of seedling growth [68]. Another gene encoding the protein ROOT INITIATION DEFECTIVE 3 was found in the vicinity of the same SNP whose homolog is involved in meristem development by acting as a negative regulator of the CUC-STM pathway in shoot apical meristem (SAM) neo-formation [40]. Two MTAs related to SDW and SLDW in chromosome 1 were found in the intronic region of a gene translating for SERINE/THREONINE-PROTEIN PHOSPHATASE 7 LONG FORM HOMOLOG/protein MAIN-LIKE 1-like. This protein has been found to be similar to protein MAINTENANCE OF MERISTEMS which may be required to maintain cell division activity in meristematic cells and contribute to plant growth (Uniprot, *Q9LNG5*, 2025).

Gene *Ca_28114* was found in the vicinity of Ca1_32048812 which was detected for RDW, SLDW and SVII in chromosome 1. It encodes for an OBERON-like protein whose homologs in *Arabidopsis* were found to encode proteins with a PHD finger domain and a coiled-coil domain. They are crucial for the maintenance and/or establishment of both the shoot and root meristems [42, 43]. GRAS proteins are a diverse family of plant-specific transcription factors having a role in plant growth & development, phytochrome signalling and stress responses [69]. Members of the GRAS transcription family include SCARECROW-like proteins that control asymmetrical cell division and endodermis differentiation to regulate ground-tissue patterning in the root. One gene encoding SCARECROW-like protein 1 was located 11 kb from MTA Ca4_38647446 on chromosome 4. The mutants of this gene has abnormal root morphology and are essential for early root development [45].

The candidate gene *Ca_04624* translates into FANTASTIC FOUR-LIKE PROTEIN whose homologs in *Arabidopsis* are responsible for regulating the size of the shoot meristem by modulating the CLV3-WUS feedback loop thereby influencing plant growth [46]. Another gene expressing HOMEOBOX-LEUCINE ZIPPER PROTEIN MERISTEM L1-LIKE was found in the vicinity of the same MTA. It has been reported to be a probable transcription factor involved in cell specification and pattern formation during embryogenesis [47].

WOX (WUSCHEL-RELATED HOMEOBOX) is a plant homeodomain-containing transcription factor (TF) family which known to play a significant role in the regulation of plant growth and development. We identified two WOX family genes that were putatively related to two other MTAs. *Ca_10316* (WUSCHEL-related homeobox 7-like) was found associated with Ca6_1942884 on chromosome 6 and *Ca_19701* (WUSCHEL-related homeobox 11) was found associated with Ca2_5629077 on chromosome 2. WOX7 mediates lateral root growth. Before the emergence of the lateral root primordia through the root surface, the WOX7 gene promoter is active in the root tips of the cortex, the endoderm initials and the endoderm. WOX11 and its closely related homolog WOX12 are recognised for their ability to regulate the development of adventitious roots—that is, roots that emerge from organs other than roots—as well as callus formation during in vitro regeneration and following damage [52].

The threshold for Bonferroni correction was reduced to include MTAs associated with our other traits (SL, RL, TSL, Germination% and SVI). We found 11 MTAs, from which we identified 5 genes associated with them to be possibly involved in ESV. *Ca_16131* was found associated with Ca2_29742379 for TSL and it translates into *Cicer arietinum* TRANSCRIPTION FACTOR MYB3-LIKE. It has been shown that MYB transcription factors are part of the early stage (days 5–15) cascade of seedling and root establishment [53]. *Ca_04390* encoding for protein ROOT PRIMORDIUM DEFECTIVE 1 was found associated with two MTAs on chromosome 4 related to TSL and SVI. This protein is involved in setting up the maintenance of active cell proliferation in advance of the establishment of the root primordium [54]. MTA Ca4_34378091 was identified by both the models for TSL and RL. One of its associated gene is *Ca_20371*, encoding SMAX-LIKE 3-like protein. Small butenolide molecules, called karrikins (KARs), have been discovered in smoke generated by burning plants. KARs have stimulating effects on seed germination, and they also modulate seedling vigour and adaptive responses that include stress acclimation, root hair growth and seedling morphogenesis. At the crossroad of strigalactone and KAR signals, SMAX1 serves as a mediator to link diverse phytohormone signalling pathways to seedling development [55]. Another gene *Ca_10366* encodes for MOTHER OF FT AND TFL1-LIKE protein. A homologue of this gene in strawberry was found to regulate seed germination and post-germination growth through integrating GA and ABA signalling in *Arabidopsis* [57]. We also identified one MTA linked to Germination percentage, and found gene *Ca_14916* linked to it. The gene is encoding AQUAPORIN TIP4-1-LIKE protein which is a possible candidate for driving the rapid cellular expansion associated with post-germination seedling growth [56].

## Conclusion

The study assessed 230 diverse chickpea genotypes for traits related to early seedling vigour in a controlled environment. The study showed a substantial amount of genetic variability of ESV traits in chickpea genotypes. High heritability of the traits implies a significant genetic basis for early seedling vigour and further possibilities in their improvement and transfer in cultivated varieties. Phenotypic screening of the association panel has helped identify highly early vigorous genotypes like ICC15567, ICC8318, ICC20259 and ICC20263, which can be utilised in the breeding pipeline for developing varieties with high ESV. In the study, we have identified 34 MTAs associated with 13 traits and 36 putative candidate genes linked to the MTAs. Out of that, we further narrowed down to 20 genes that could potentially be directly or indirectly involved in providing ESV to chickpea. From the function of associated genes, it is apparent that genes related to meristem function (LOC101500401), root formation (LOC101514996), and auxin signalling (LOC101510474) may play an important role in governing ESV in chickpea. Genes identified through our study need to be validated further through functional genomic approaches in order to gain a better understanding of their functions. The findings presented in our study will help in further genetic improvement and breeding for cultivars having high ESV and suitable for rice-fallow lands.

## Supplementary Information

Supplementary Material 1.

Supplementary Material 2.

## Data Availability

The genotypic dataset analysed in this study is available in the NCBI Bioproject under accession number PRJNA657888. The phenotypic dataset supporting the conclusions of this article is provided in the supplementary materials.
