# Supplementary material for: Dissection of genomic regions underlying early seedling vigour in chickpea through genome-wide association mapping
Source: BMC Plant Biol. 2025 Dec 5;26:55. doi: 10.1186/s12870-025-07814-x (PMC12797933; doi:10.1186/s12870-025-07814-x)
Supplement: Supplementary file 2 — Supplementary Material 2. [file 12870_2025_7814_MOESM2_ESM.docx]

**Supplementary file-** Genomic Dissection of Early Seedling Vigour Traits in Chickpea Reveals Key Marker-Trait Associations" (New revised title: Dissection of Genomic Regions Underlying Early Seedling Vigour in Chickpea through Genome-Wide Association Mapping) (Submission ID: e182e59a-e1f6-49f3-884d-adf9543dc6c1).

**
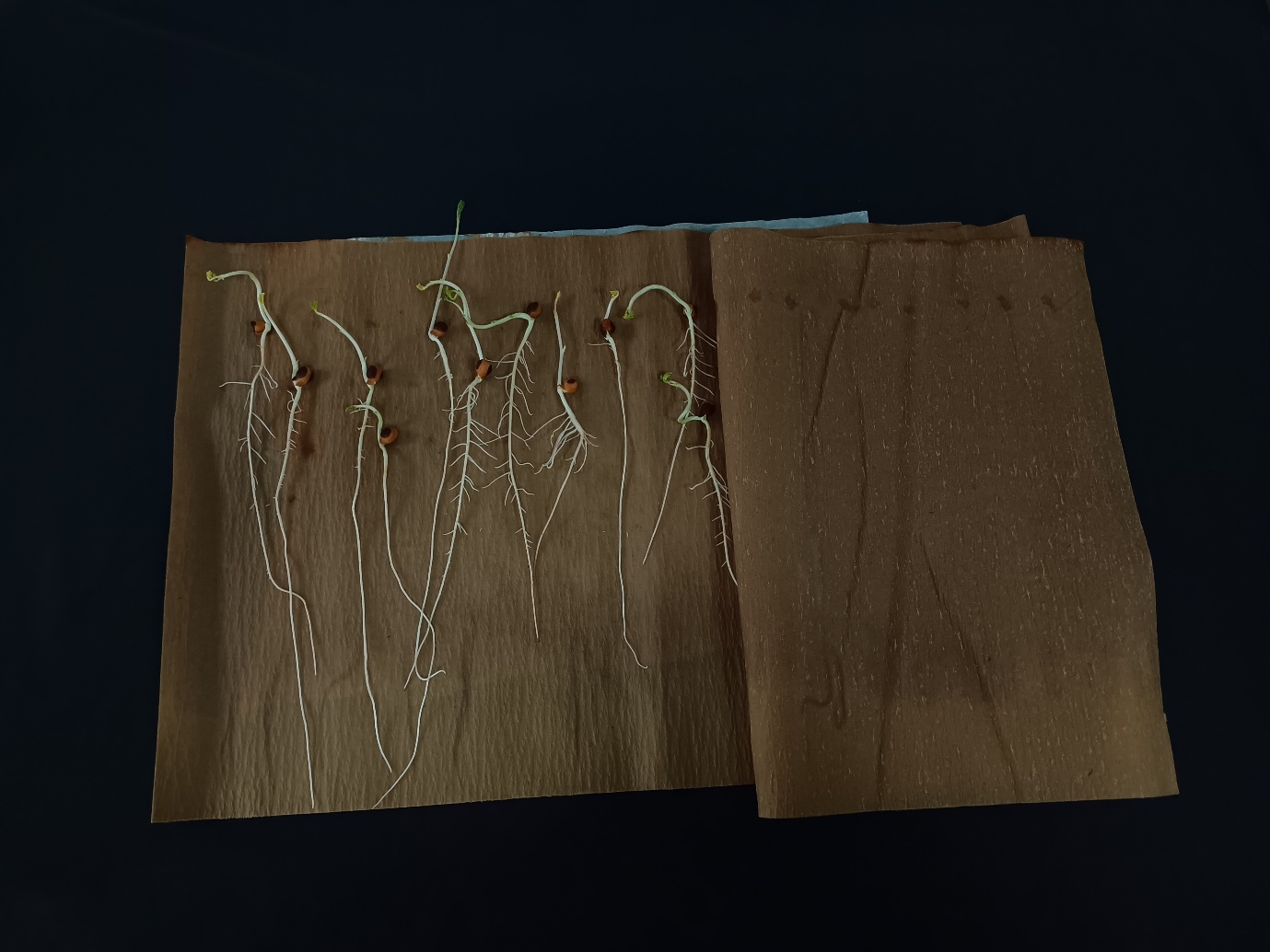
**

**Supplementary Fig. S1. Representative image of chickpea seedlings used for phenotyping root length, shoot length, and early seedling vigour traits.**

**
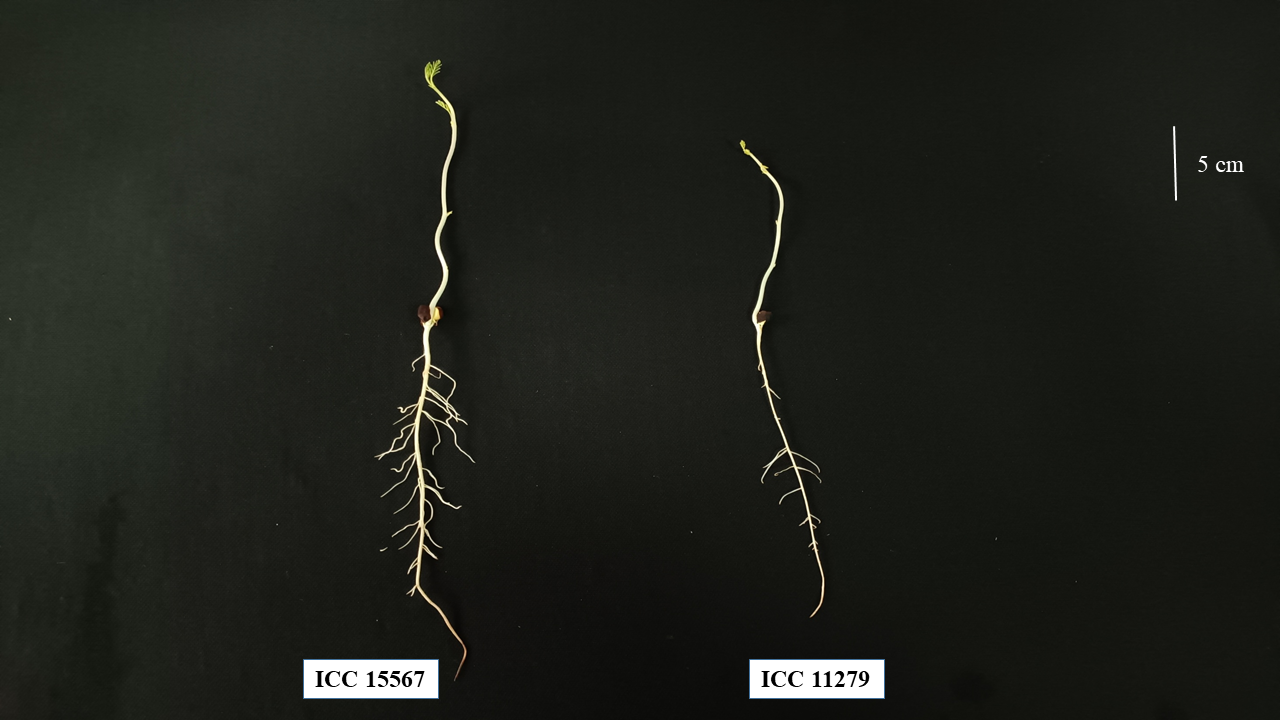
Supplementary Fig. S2. Representative image of chickpea seedlings displaying contrasting phenotypes for root and shoot growth at the early seedling stage.**


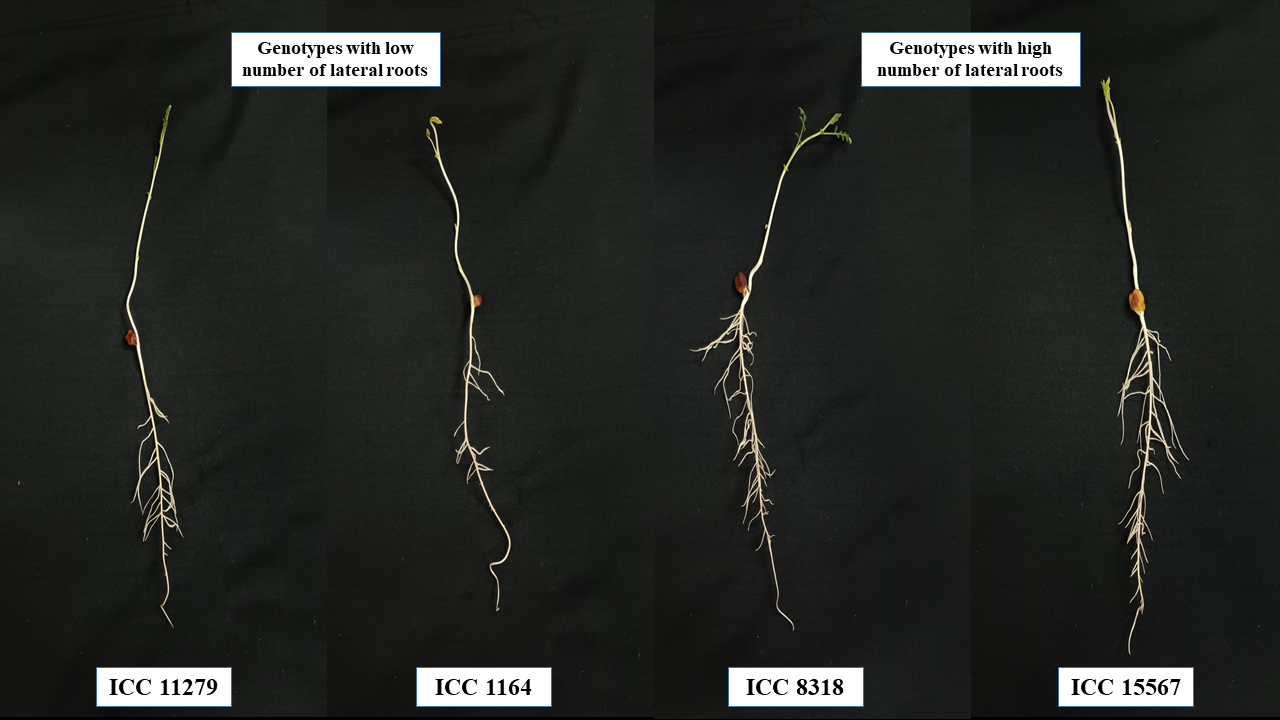


**Supplementary Fig. S3. Representative photos of chickpea genotypes with a variable number of lateral roots**

**
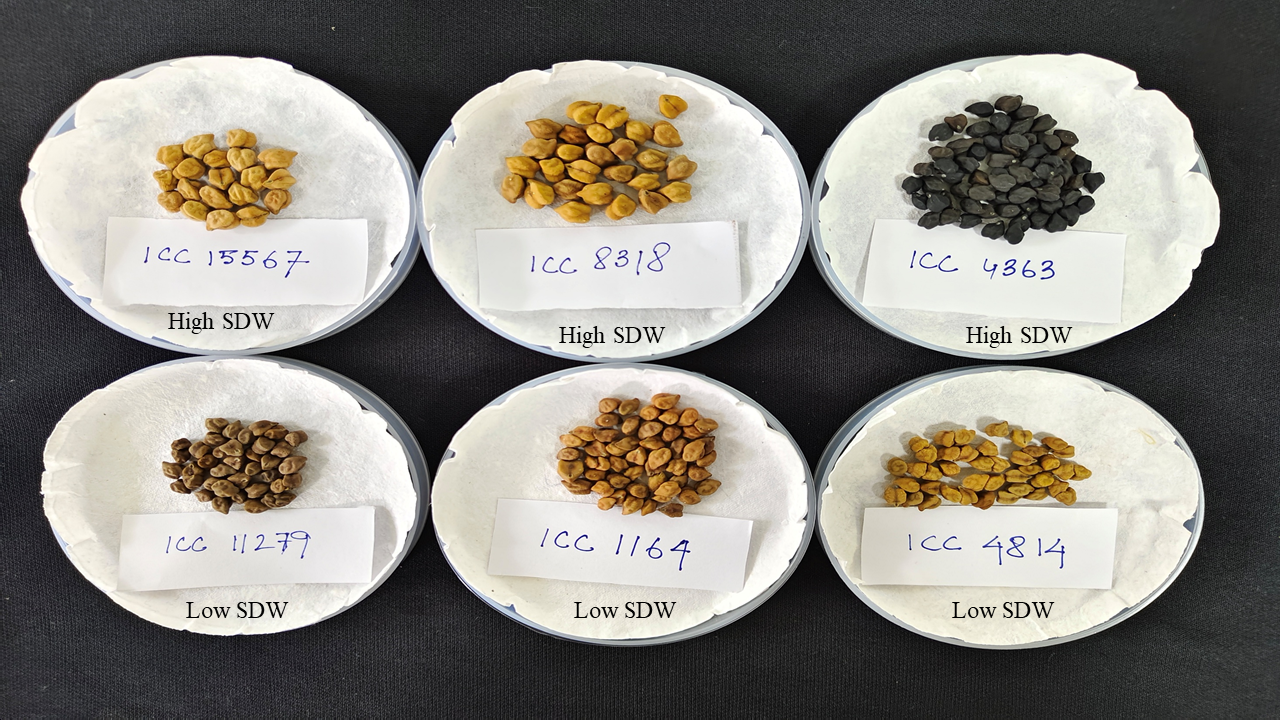
**

**Supplementary Fig. S4. Seed images of higher SDW and lower SDW chickpea genotypes**


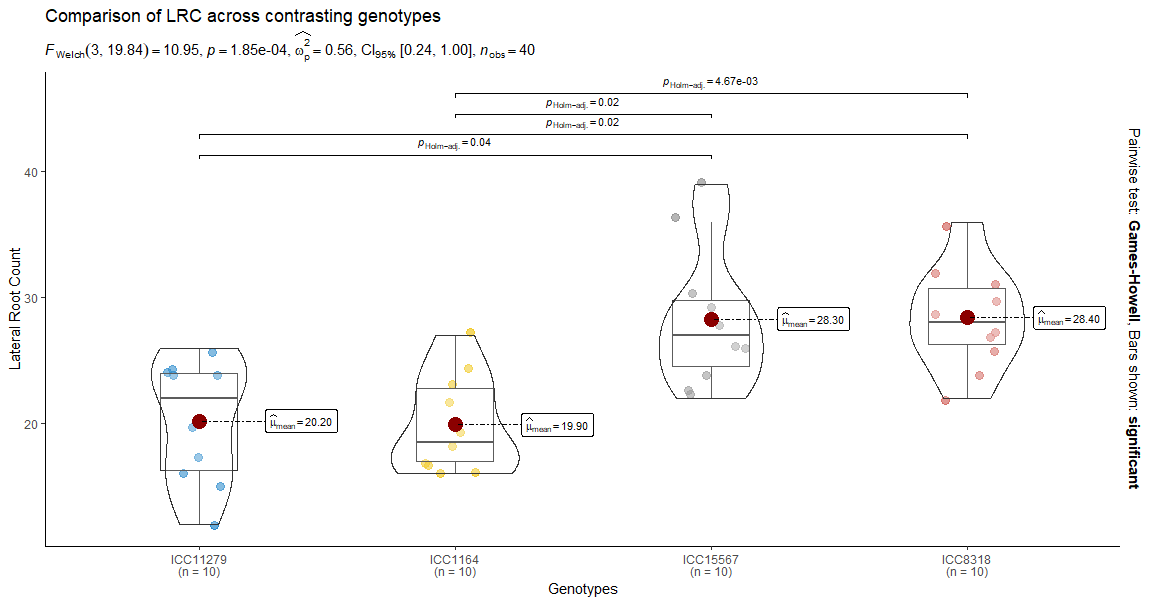


**Supplementary Fig. S5.** Comparison of lateral root count (LRC) among the four contrasting chickpea genotypes at the early seedling stage. Lateral roots having a length >1 mm were considered in the count. Genotypes ICC15567 and ICC8318 showed significantly higher LRC values, whereas ICC11279 and ICC1164 exhibited lower LRC, confirming phenotypic differences consistent with genetic variation in lateral-root–associated genes identified in the study. Data represent mean ± SE (n = 10), and statistical significance was determined by one-way ANOVA followed by Games–Howell post-hoc test (*p* < 0.05).
